# Hints to Explorers and Naturalists in the Field

**Published:** 1890-02

**Authors:** R. W. Shufeldt


### HINTS TO EXPLORERS AND NATURALISTS IN THE FIELD, ABOUT THE PREPARATION, CARE AND TRANSPORTATION OF VERTEBRATE SKELETONS IN THE ROUGH.

By R. W. Shufeldt, M.D., C.M.Z.S.

To those who have watched the progress of the zoölogical sciences during the past few years, it must be evident that the subject of morphology is entering more and more each year into our systems of classification of the forms upon the earth.

It is none the less evident, even so far as the extensive vertebrate fauna of this country is concerned, that the first settling of its hundreds of representatives in their places in the system, as well as their superficial characters will permit, has about been accomplished. Now, although such a classification roughly meets the requirements of the orderly mind in allowing it to survey almost at a glance the varieties, kinds and number of animals in the United States, we must know that this classification is yet largely provisional. The more intimate knowledge of the relationship of forms and their final classification is the work of the years before us, and will be based upon our digested understanding of the structure of all forms—comparative morphology—and morphology in its entirety as worked out for each individual.

In vertebrate morphology, osteology has always held a very important place. The reason for this must be patent to every one. It is the skeleton that really supports the form of the animal; gives attachment to the vast majority of its muscles; protects its various viscera and soft parts; and constitutes the remains of animals that have lived in former ages. So the subject of the study of skeletons, and more particularly the comparative study of skeletons, comes to be one of the highest importance.

Many of the skeletons that drift into our museums or fall into the hands of those who are engaged in describing them and elucidating the significance of the characters they present, are collected by naturalists accompanying expeditions, or by explorers, or by paid collectors.

Very often such skeletons are sent to me for description or to assist me in my osteological work, and I have as frequently observed that these specimens are incomplete in many important particulars. My observation teaches me that this incompleteness is due in nearly every instance to the inconversance with the skeleton on the part of the person who prepared it, rather than to his carelessness.

Under these circumstances I must believe that the hints I offer below cannot come amiss, and if borne in mind by those who make skeletons in the rough, and put into practice by explorers generally, cannot fail but be of great value in the end to all concerned.

Before preparing the skeleton of any vertebrate we may have in hand, it is of *prima facie* importance for us to know the correct name of the animal as given in the most recent classification. In other words we must be sure of the identification of our specimen.

If in the field, the most convenient place to prepare skeletons is near a running stream of clear water, though it is often necessary to resort to other places ; at any rate we must, if possible, have plenty of clean water at hand.

The instruments for our work are few and simple. They consist essentially of a pair of blunt-pointed scissors, a sharp dissecting scalpel, a brush, like an ordinary nail-brush such as we use in the toilet, and, finally, a coarse knitting needle, will be found useful in many cases.

Let our specimen be some bird of a medium size, recently killed. The first step consists in carefully removing all the integuments, from base of beak to suffrago, and from tip to tip of manus. Of course, this operation does not call for the care we take in removing a skin for taxidermical purposes ; the skin may be picked up anywhere and removed in pieces. Next we give our specimen a good washing in the water until all blood ceases to flow and the muscles look quite pale. If any of the limbs have been fractured completely across, these may now be removed by dividing their soft attachments, and set aside to be cleaned subsequently. Next, eviscerate the specimen by *carefully* cutting through the thin walls of the abdomen. Care must be exercised here not to fracture any of the ribs or delicate bony processes of the vertebræ or other bones. In removing the lungs we must leave the parts that ossify, such as belong to the respiratory apparatus, as the trachea or large bony box forming the larynx, among the *anatidæ*. Again we thoroughly wash our specimen until all the blood has disappeared resulting from this operation.

We now devote our attention to the head. The horny sheath of the bill is always allowed to remain, notwithstanding it in no way belongs to the skeleton proper. In the eyes of all birds and many other vertebrates, as lizards and fishes, there exists a circlet of bony plates, known as the sclerotals. These are best preserved by allowing the eyes to remain in the specimen. The ball of the eye may, however, be tapped by a small incision through the pupil and the vitreous humors allowed to escape. The mistake is often made of removing the eyes altogether and throwing them away. Another, and if possible the more frequent and yet graver, error is committed in removing the tongue and its bony support, the "hyoid arches." Even in the prepared skeletons of the largest of mammals this bone is nearly always missing. These hyoid bones may be seen curving up behind the occiput in any bird, and should *always* be allowed to remain in our preparation of a skeleton in the rough. This caution applies also to all other vertebrates where these bones occur.

In birds we frequently find small sesamoids about the head, as at the angle of the jaw ; these, with certain bony appendages occurring about the eye, as in many diurnal *raptores*, should be carefully attended to, and exercising great care in regard to the delicate lacrymal bone in all cases, we may be pretty sure of having retained the skeleton of the head and its associate parts. The bellies of the largest muscles may be carefully cut away, but no harm is done by allowing the brains to remain in the cranial cavity. If we suspect any of the appendages about the head contain osseous supports, they must receive our special attention.

The skeleton of the neck from base of skull to the first pair of ribs that join with the sternum, can be allowed to dry along with the bony trachea (birds) just as it is, after removing the skin. If our time permits, however, it is better to carefully cut away all the muscle we can, being watchful of the delicate vertebral processes in many birds and animals. Among the former, the first pair of ribs are usually very delicate and freely suspended (see Fig. 35 in my "Osteology of Eremophila," Hayden's 12th Annual), and in cormorants we find a bony style at the occiput. Many free bones occur in the necks of other vertebrates, and especially in the young, so in case we are ignorant of the osteology of the form we are preparing, we must exert unusual care, and be on the alert to pay the proper attention to the slightest warning from the edge of our scalpel.

In cleaning the body and limbs the general rule to be applied, is to carefully elevate each muscle with the handle of the scalpel, dividing it with the scissors *just* beyond its tendons of origin and insertion, through the fibres. In this way we are pretty safe in leaving ossified tendons, sesamoids, and other bony parts. Sesamoids, such as the patella, the os prominens, the os humero scapulare (at the shoulder in birds), the sesamoids at the elbow in certain auks, and fingers, toes, claws and many other bony parts must be carefully left in their places. The muscles between the ribs in large animals can be carefully removed, but in smaller subjects they are best allowed to remain, as they serve to protect this delicate framework as well as to preserve the form of the specimen.

The caudal appendage is treated much in the same way as advised in speaking of the neck. It may have many free bones attached to it, that require to be left, holding their normal relations to other osseous parts as well as they may.

Fishes, of course, require, if possible, greater care on the part of the preparator, and the constant exercise during the operations of all his ingenuity and knowledge of the anatomy of the form before him.

After all the soft parts have been properly removed, the skeleton of the subject must be once more carefully washed in clean water until all signs of flowing blood disappear. The remnants of muscles and other parts clinging to the bones may be induced to come off by a careful use of the brush, and in any event this will be found an efficient aid in giving the skeleton a cleanly appearance. Broken bones must be carefully splinted and wrapped with thread or twine. Bones that are independent and must of necessity come away, must be carefully cleaned, wrapped in gauze netting, together with any notes on a slip of paper that may be required to perfectly describe their relation to the specimen, and the whole parcel tied securely to the main skeleton.

It remains now for us to do our subject up in a convenient bundle, properly label it, and place it in a position for drying. In small subjects, good, strong, soft twine is the best thing to wrap them up with. Some birds admit of having the head and part of the neck carefully introduced into the empty cavity of the thorax. The practice has some disadvantages, however, when the mechanical osteologist comes to mount the specimen, as the ligaments harden in an awkward position. The limbs should be drawn up close to the sides of the body, making the same angles, only more acute as the muscles are gone, as they would in nature.

They may be secured in these positions by tying to the pelvis or shoulder girdle, choosing the strongest bones in both cases. In birds and such forms as the ornithorhynchus, the podotheca should be allowed to remain, as it protects the toes and preserves the form of the foot to a large extent.

Our skeleton should now be finally secured by passing the twine round it a sufficient number of times to render all the bones more or less immovable, and tie the ends.

Next, the specimen should have a full and intelligent label attached to it. This must be made fast to one of the long bones or, as in fishes, to the vertebral column, and *not*, as I have so often seen in specimens, punched through the interorbital septum in birds or to the delicate styloid process in mammals.

The label should aim to record the place and time of capture ; the name of the specimen ; collector and preparator ; the sex ; the probable age ; accidents to the bones that have occurred either incident to its capture or subsequent preparation ; and finally, calling attention to any special bones that have been noticed, or are attached in a separate parcel accompanying the specimen.

A duplicate of this label should be kept in the explorer's note book.

The specimen must now have a separate loop of cord attached to it and be hung up in a safe place to dry. By safe, I mean where vermin of any description will be unable to reach it, or where it will not be subject to other injury.

The time for drying depends upon the size of the animal, the climate, and the time of year, from two to several days.

In packing for transportation, it is well to fill all cavities, such as the chest, with the material known as "imperial," or with fine, dry hay, or if in the South, the moss known as Spanish beard, so common there. Of all these materials, however, the imperial is the best, if available, both for filling the cavities of the animal, and the general packing afterward.

In concluding, the writer is well aware that there is a great deal more that could be said upon this highly important subject. He trusts, however, that these remarks will, by reaching the proper persons, have the effect of inducing them to exercise a better care in the preparation of vertebrate skeletons in the field, and by not destroying or throwing away such parts as the hyoid, the sclerotals and sesamoids, as is almost universally done, enable them to bring or send home skeletons of greater value to the descriptive anatomist or to those in charge of museums of anatomy.

